# Molecular Characterization of Atypical Hepatitis B Serological Profiles in HBsAg-Negative Women of Childbearing Age in Gabon

**DOI:** 10.3390/cimb48020184

**Published:** 2026-02-06

**Authors:** Ismaël Pierrick Mikelet Boussoukou, Aude Sandrine Andeme Eyi, Jean Alban Ondh-Obame, Philippe Jacques Nathanaël Ondamba, Marien Juliet Magossou Mbadinga, Opheelia Makoyo Komba, Serge Thierry Omouessi, Joel Fleury Djoba Siawaya, Bénédicte Ndeboko

**Affiliations:** 1 Laboratoire du Centre Hospitalier Universitaire Mère-Enfant Fondation Jeanne Ebori, Libreville BP 212, Gabon; mikeletpierrick@gmail.com (I.P.M.B.); eyiaude@gmail.com (A.S.A.E.); n.ondamba@gmail.com (P.J.N.O.); julverald@gmail.com (M.J.M.M.); joel.djoba@gmail.com (J.F.D.S.); 2 Département de SVT, Ecole Normale Supérieur, Libreville BP 17009, Gabon; ondhobame@gmail.com; 3 Service de Gynécologie du Centre Hospitalier Universitaire Mère-Enfant Fondation Jeanne Ebori, Libreville BP 212, Gabon; makoyokombaopheelia@gmail.com; 4 Département de Physiologie, Faculté de Médecine, Université des Sciences de la Santé, Libreville BP 4009, Gabon; omouessithierry@gmail.com; 5 Département de Biologie Cellulaire & Moléculaire, Génétique, Faculté de Médecine, Université des Sciences de la Santé, Libreville BP 4009, Gabon

**Keywords:** hepatitis B, atypical serological profiles, HBsAg-negative, real-time PCR, occult HBV infection, women of childbearing age, molecular diagnostics

## Abstract

Occult hepatitis B infection (OBI) and mutated forms of the hepatitis B virus (HBV) represent diagnostic challenges, especially in individuals with atypical serological profiles. This study explores the molecular characteristics of HBV in HBsAg-negative women of childbearing age exhibiting atypical serological markers. We selected 100 HBsAg-negative sera from a cohort of 433 women aged 15–45 years. Additional HBV serological markers (anti-HBc, anti-HBs, HBeAg, anti-HBe) were assessed. Real-time PCR targeting the HBV S gene was performed on samples presenting atypical profiles. Socio-demographic and clinical correlates were also analyzed. Atypical serological profiles were identified in 23% of HBsAg-negative women, including combinations such as isolated anti-HBe positivity and anti-HBe with anti-HBc. Among these, none tested positive for HBV DNA by real-time PCR. Atypical profiles were more prevalent among women attending antenatal consultations and those aged under 25 years. The absence of detectable HBV DNA suggests either very low viral loads, resolved past infections, or serological artifacts due to mutated HBV strains. The high frequency of atypical serological patterns among HBsAg-negative women underscores the need to refine molecular diagnostic tools for detecting occult or mutated HBV. Further sequencing and genotypic characterization studies are warranted.

## 1. Introduction

Hepatitis B virus (HBV) infection remains a major public health concern worldwide, with an estimated 296 million chronic carriers and approximately 820,000 annual deaths due to HBV-related complications, including cirrhosis and hepatocellular carcinoma [1,2]. In women of childbearing age, HBV poses a dual threat—first to the health of the mother, and second to the unborn child due to the risk of vertical transmission. Consequently, accurate and early diagnosis of HBV infection in this population is crucial for effective prevention and management strategies [3,4].

Conventionally, HBV infection is diagnosed by detecting the hepatitis B surface antigen (HBsAg), the hallmark marker of active infection. However, a subset of individuals may exhibit atypical serological profiles—i.e., patterns that do not conform to classical interpretations—despite being HBsAg-negative. These patterns may include isolated anti-HBe positivity, the presence of anti-HBc with or without anti-HBs, or discordant combinations of other HBV markers. Such profiles are frequently encountered in clinical practice but are poorly understood and often overlooked [5,6,7].

Atypical profiles can reflect various underlying conditions, including resolved past infections, false-positive results, or occult hepatitis B infection (OBI). OBI is defined by the presence of HBV DNA in the liver (and sometimes in the blood) of individuals who test negative for HBsAg, with or without the presence of anti-HBc or anti-HBe [8,9]. OBI is particularly concerning in transfusion settings, immunocompromised individuals, and pregnant women, due to the potential for viral reactivation or vertical transmission [10,11,12].

The occurrence of atypical serological patterns in women of reproductive age, especially during pregnancy, warrants closer investigation. Limited data exist on the molecular characterization of such profiles in this specific population [13,14]. This study aims to explore the serological and molecular patterns of HBV in HBsAg-negative women of childbearing age, with a focus on identifying possible occult infections or infections involving mutant HBV strains that may escape routine detection [15,16,17].

Through targeted real-time PCR analysis of sera with atypical HBV profiles, this study seeks to contribute to a better understanding of HBV pathogenesis and diagnostic limitations, and to inform strategies for more accurate detection of non-classical forms of HBV infection [18,19].

## 2. Materials and Methods

### 2.1. Study Design and Population

This was a laboratory-based, cross-sectional exploratory study conducted on a subset of women of childbearing age previously enrolled in a broader sero-epidemiological survey on hepatitis B virus (HBV) in Gabon. From the initial cohort of 433 participants aged 15–45 years, 100 HBsAg-negative sera were randomly selected by a simple random sampling method to ensure representativeness in further serological and molecular analysis. The selected subset included 50 samples from pregnant women and 50 from non-pregnant women, chosen to provide balanced representation for comparative analysis.

The random selection of 50 pregnant women was not stratified by trimester at the time of sampling; however, trimester information was retrieved retrospectively from clinical records and used for secondary analysis.

### 2.2. Serological Testing

All selected sera were screened for HBsAg and other hepatitis B virus (HBV) serological markers—including anti-HBs, anti-HBc, HBeAg, and anti-HBe—using rapid diagnostic tests (RDTs), specifically the HBsAg Rapid Test Cassette (serum/plasma) and the HBV Combo Check kit (Medic Expert, Nancy, France). Atypical serological profiles were defined as isolated or discordant marker patterns, such as the presence of anti-HBe in the absence of other HBV markers. Due to reagent limitations, only 100 HBsAg-negative samples were randomly selected for extended serological testing. These samples were stratified into two groups: 50 from pregnant women and 50 from non-pregnant women. All assays were performed in accordance with the manufacturers’ instructions.

### 2.3. HBV Serological Testing and Assay Performance

HBsAg detection was first carried out using the Determine™ HBsAg rapid diagnostic test (RDT) (ALERE, Abbott Point of Care, Abbott Park, IL, USA), which has a reported sensitivity of 96.4% and a specificity of 100%. All results obtained with the RDT, whether positive or negative, were subsequently confirmed using the miniVIDAS^®^ ELISA system (bioMérieux, Lyon, France), in accordance with the manufacturer’s instructions.

In addition, all HBV markers (HBsAg, anti-HBs, HBeAg, anti-HBe, and anti-HBc) were assessed using the HBV Combo Check (Medic Expert, Nancy, France), a rapid chromatographic immunoassay that simultaneously detects five HBV markers in serum or plasma. The reported performance characteristics are as follows:

• HBsAg: sensitivity > 99.9%, specificity 99.4%, accuracy 99.7%
• Anti-HBs: sensitivity 96.5%, specificity 97.8%, accuracy 97.3%
• HBeAg: sensitivity 96.3%, specificity 97.9%, accuracy 97.5%
• Anti-HBe: sensitivity 97.3%, specificity 97.9%, accuracy 97.7%
• Anti-HBc: sensitivity 97.8%, specificity 97.7%, accuracy 97.8%

Both assays demonstrate high diagnostic reliability, consistent with quality standards for epidemiological and clinical investigations, enabling the detection of both typical and atypical HBV serological patterns with minimal error rates. To further ensure reliability, all HBsAg results (positive or negative) were confirmed using MINI VIDAS^®^ (Biomérieux, Lyon, France), a compact automated immunoassay platform based on the enzyme-linked fluorescent assay (ELFA) principle. Atypical serological profiles were defined as discordant combinations, such as isolated anti-HBc or anti-HBe positivity in the absence of HBsAg.

### 2.4. Molecular Analysis

#### 2.4.1. DNA Extraction

Viral DNA was extracted from 400 µL of the sample using the Exiprep™ 16 Dx automated system (Bioneer, Daejeon, Republic of Korea) and the Exiprep™ Dx Viral DNA/RNA Kit, following the manufacturer’s instruction. This system is optimized for high-throughput purification of viral nucleic acids from clinical samples.

#### 2.4.2. Real-Time 20 Amplification

Detection of HBV DNA was performed by real-time polymerase chain reaction using the AccuPower^®^ HBV Quantitative PCR Kit (Bioneer, Republic of Korea) on the Exicycler™ 96 Real-Time Quantitative Thermal Block, following the manufacturer’s instructions. This assay is based on TaqMan probe chemistry and is designed to detect conserved regions of the HBV genome with high specificity and sensitivity. The primers and probes used target the 5′ region of the gene coding for HbsAg.

Each PCR reaction was prepared using lyophilized premix reagents provided in the kit, which include HBV-specific primers, a fluorescent-labeled probe, Taq DNA polymerase, dNTPs, MgCl_2_, and a buffer system. A volume of 5 µL of the purified DNA eluate was added to each reaction tube. The amplification protocol included an initial denaturation at 95 °C for 5 min, followed by 40 cycles of 95 °C for 15 s and 55 °C for 45 s.

Internal controls were included in each reaction to validate amplification efficiency and detect any potential inhibition. Negative and positive controls were also run in parallel to ensure assay reliability. Fluorescence data were collected in real time, and results were interpreted according to the cycle threshold (Ct) values. A Ct value < 38 was considered positive for HBV DNA.

#### 2.4.3. Molecular Assay Performance and Sensitivity

The AccuPower^®^ HBV Quantitative PCR Kit (Bioneer, Republic of Korea) used in our study has a limit of detection of 20 IU/mL as indicated by the manufacturer. Internal positive and negative controls were included in all runs to validate amplification efficiency.

### 2.5. Ethical Considerations

This study was approved by the General Management and Scientific Council of CHUME-FJE on 20 May 2022 (Approval ID: CHUME-FJE/008/22/05/20). All procedures involving human participants were conducted in accordance with the ethical standards of the institutional research committee and with the 1964 Declaration of Helsinki and its later amendments or comparable ethical standards. Written informed consent was obtained from all participants, with or without the presence of a family witness. Participant anonymity and data confidentiality were maintained throughout the study.

### 2.6. Statistical Analysis

All statistical analyses were conducted using Stata software, version 15. Continuous variables were reported as means with standard deviations when normally distributed, as determined by the Shapiro–Wilk test. Categorical variables were compared using Pearson’s chi-square test or Fisher’s exact test when expected cell counts were low. A *p*-value of less than 0.05 was considered statistically significant.

## 3. Results

### 3.1. The Flow Diagram

**Figure 1:**
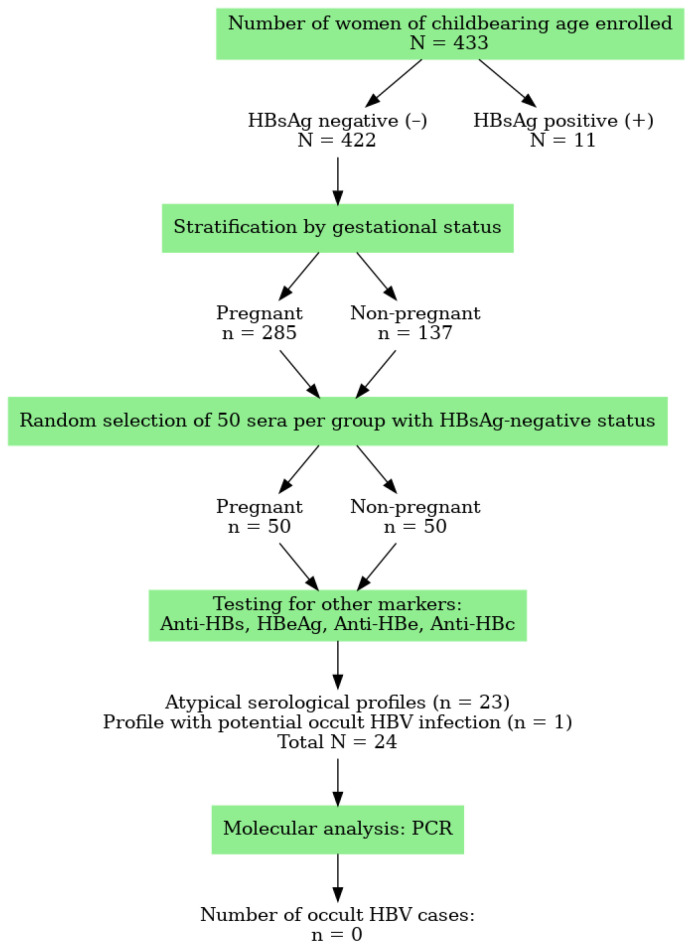
Flowchart of inclusion and diagnosis of occult hepatitis B.

The flow diagram below shows the number of women/sera according to the inclusion and diagnostic procedure for occult hepatitis B in our study at CHUME-FJE (Figure 1).

### 3.2. Prevalence of Atypical Profiles

Among the samples testing negative for hepatitis B surface antigen (HBsAg), only 100 were randomly selected for molecular analysis due to reagent availability constraints and on these samples HBsAg-negative women, 23 (23%) (CI 95%: 15.4–32.7) exhibited atypical HBV serological profiles (Figure 1). A higher frequency was observed in pregnant women (n = 14 or 28%) (CI 95%: 16.7–42.7) compared to non-pregnant women (n = 9 or 18%) (CI 95%: 9.0–31.9), although this difference was not statistically significant (*p* = 0.411).

The most frequently observed atypical pattern was the presence of anti-HBe in isolation (n = 14), followed by the co-occurrence of anti-HBe and anti-HBc without detectable anti-HBs or HBsAg (n = 9). Additionally, no woman had isolated anti-HBc antibodies (Table 1).

In addition, among the seven samples not molecularly tested, two showed invalid marker combinations that could not be reliably interpreted, while the remaining five were consistent with seroconversion profiles, suggesting recovery from past HBV infection rather than occult infection (Table 1).

### 3.3. Concordance Between the Rapid Diagnostic Tests and the miniVIDAS^®^ ELISA

The overall agreement was high (100%), with no discordant results observed between the Rapid Diagnostic tests and the miniVIDAS^®^ ELISA, confirming the reliability of our serological testing. In over words, “no discordant HBsAg results were observed between the two methods”.

### 3.4. Distribution According to Gestational Trimester

**Figure 2:**
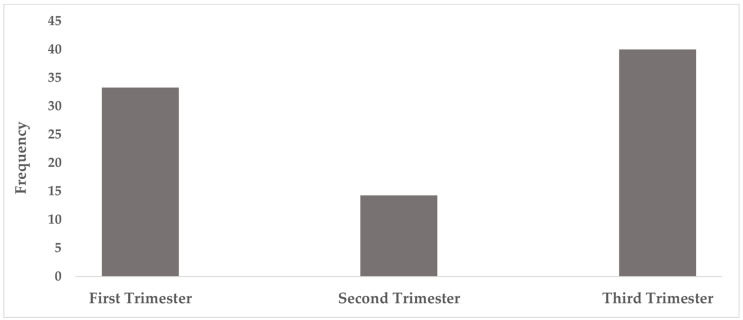
Among pregnant women, the frequency of atypical serological profiles was higher in those in the third trimester 50% (n = 7) and the first trimester 35.7% (n = 5) of pregnancy.

Among pregnant women with atypical serological profiles, the distribution by trimester showed a predominance in the third trimester (50%) (CI 95%: 23.6–76.3), followed by the first trimester (35.7%) (CI 95%: 14.4–58.8), and lowest in the second trimester (14.3%) (CI 95%: 3.8–37.4). However, no statistically significant association was observed between trimester and atypical profile frequency (*p* = 0.287) (Figure 2).

### 3.5. Sociodemographic and Clinical Associations

The study cohort (n = 100) included women aged 15–45 years, of whom 30% were under 25 years, 90% were single, and 10% were married. In terms of occupation, 45% were students, 19% unemployed, and 36% employed or craftswomen. Regarding reason for hospital consultation, 37% attended for antenatal care and 62% for biological testing.

Atypical serological profiles were more frequently observed among women aged < 25 years (26.7%) and among single women (24.4%) compared to their older and married counterparts. Although not statistically significant, students (28.9%) and unemployed (26.3%) women were more likely to exhibit atypical profiles than employed women (13.3%) or craftswomen (16.7%).

A significant association was found between reason for hospital consultation and the presence of atypical profiles (*p* = 0.028). Women who consulted for prenatal care were more likely to show atypical patterns (32.4%) compared to those who came for biological testing (16.1%). In addition, no participants presented symptoms suggestive of HBV complications, as all were asymptomatic outpatients.

Overall, the multivariate analysis did not reveal any factors independently associated with the atypical HBV profile.

### 3.6. Molecular Detection of HBV DNA

**Figure 3:**
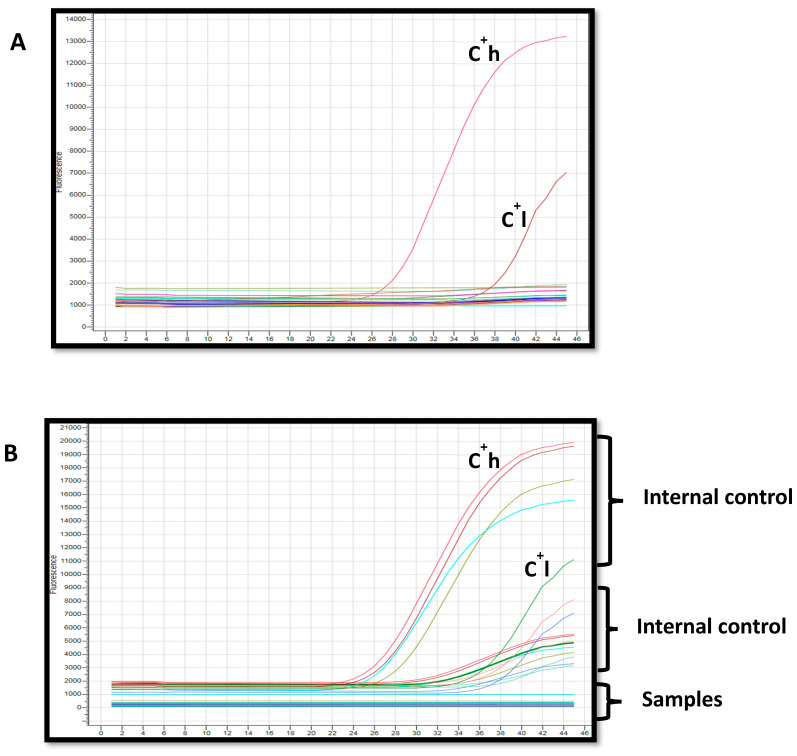
Real-time PCR results. (**A**): Internal controls; C^+^h (high positive control), C^+^l (low positive control); (**B**): Internal positive controls contained in the samples and negative samples are represented by horizontal and linear lines.

Of the 23 sera with atypical serological profiles, all were tested by Real Time PCR for the presence of HBV DNA. Surprisingly, none of the samples tested were positive for HBV DNA, indicating undetectable viremia in this population subset (Figure 3).

## 4. Discussion

Our study revealed a circulation of atypical serological profiles among HBsAg-negative women of childbearing age in Gabon, with a predominance in pregnant women, although this difference was not statistically significant. This finding is consistent with the growing body of literature indicating that atypical or occult hepatitis B virus (HBV) infections (OBIs) represent a silent but significant public health concern, especially in endemic regions like sub-Saharan Africa [6,10].

Interestingly, while atypical HBV profiles have been reported elsewhere in Africa, the 23% prevalence observed in Gabon is comparatively high and represents the first molecularly assessed report among HBsAg-negative women of childbearing age in this setting. The association with young and pregnant women highlights a new public health concern, emphasizing the need for strengthened antenatal HBV screening and clinical vigilance in Gabon.

Atypical profiles, particularly isolated anti-HBe or anti-HBc positivity, may reflect either a resolved past infection, serological window phases, or occult hepatitis B infection (OBI), where HBV DNA may persist in the liver or blood without detectable HBsAg [8]. Although our molecular tests did not detect HBV DNA in the atypical samples analyzed, this does not entirely exclude the possibility of low-level or transient OBIs, particularly given the sensitivity limits of our assays and the known fluctuation of HBV DNA levels in OBI cases [20].

HBsAg−/Anti-HBs−/HBeAg+/Anti-HBe−/Anti-HBc− profile is not typical of occult HBV infection. Although rare, isolated HBeAg positivity may be explained by mutations in the S gene leading to defective or undetectable HBsAg production, while viral replication persists and maintains HBeAg expression. It could also reflect a low-level or mutant HBV infection producing minimal antigen concentrations below the detection threshold of conventional assays. Such mechanisms have been described in previous studies investigating atypical or occult HBV infections [5].

As for isolated HBsAg, we can explain it by transient immune responses following partial viral clearance, mutations in the precore or basal core promoter regions reducing HBeAg expression, or host immune modulation—particularly during pregnancy—leading to atypical serological responses.

Previous studies have demonstrated that OBI may still occur in the absence of detectable viremia, especially in peripheral blood, with viral genomes often confined to hepatic tissue [11]. Furthermore, certain viral variants or mutations in the S gene—particularly within the ‘a’ determinant region—can alter the antigenicity of HBsAg and result in false-negative results on routine assays, thereby contributing to atypical serological patterns [5,15].

The detection of genotype A as the most prevalent strain in previous studies in Gabon [21] and its known association with chronicity and hepatocarcinogenesis [19] highlights the importance of molecular surveillance in this region. Although our study did not genotype HBV strains due to the absence of detectable DNA, the high prevalence of atypical profiles suggests the need for continued molecular investigations, especially among pregnant women.

The presence of isolated anti-HBe or anti-HBc antibodies—common in our cohort—could represent immune memory from past infection, could also conceal low-level viremia, window periods, or even low-grade replication in the setting of immune escape variants [16,18]. These profiles are clinically relevant, particularly in pregnant women, where incomplete detection may fail to identify risks for perinatal transmission.

While no HBV DNA was detected in atypical profiles, we believe the serological findings remain scientifically relevant. They demonstrate the high frequency of atypical profiles in HBsAg-negative women in Gabon, which is epidemiologically important even if molecular confirmation was negative. The lack of DNA detection could reflect (i) very low-level occult infection below the detection threshold, (ii) resolved past infections.

Importantly, the 20 IU/mL detection limit of the AccuPower^®^ kit may not be sufficiently sensitive to detect very low or fluctuating HBV DNA levels typical of occult infections, which could explain the negative results.

All of the conditions mentioned above can promote the reactivation of hepatitis B infection in immunocompromised pregnant women, potentially increasing the risk of vertical transmission.

In addition, it is important to note that among the seven cases not molecularly tested, two displayed invalid marker combinations, while the remaining five were consistent with seroconversion patterns, indicating potential recovery from past infection. These profiles, being more compatible with resolved HBV infection than with atypical or occult infection, were therefore not included in the molecular testing. This methodological choice may have led to an underestimation of anti-HBs–positive atypical profiles, but it allowed us to focus our PCR analysis on patterns more suggestive of occult HBV infection.

Moreover, a limitation of our study is that only 23 sera were tested by PCR, and HBV DNA detection in occult cases is known to be intermittent [22,23]. This restriction may have underestimated the true prevalence of OBI in our cohort, highlighting the need for larger studies with repeated nucleic acid testing and, when feasible, liver tissue analysis.

Given these findings, our results underscore the importance of improving diagnostic strategies by integrating both serological and molecular tools in HBV screening, particularly in antenatal care settings. In resource-limited contexts, a targeted approach to molecular testing based on atypical serological patterns could optimize resource use while improving detection and intervention.

Taken together, although no HBV DNA was detected in our study, the high prevalence of atypical serological profiles among HBsAg-negative women of childbearing age suggests the potential for underdiagnosed or misclassified HBV infections. These findings advocate for further studies incorporating more sensitive molecular techniques and liver tissue analysis to uncover the true burden of occult and variant HBV infections in endemic populations.

### 4.1. Limits of This Study

Major limitations of our study are the small sample size, which may reduce statistical power and generalizability of the findings, and the absence of liver biopsy, which remains the gold standard for confirming occult hepatitis B infection (OBI) through intrahepatic HBV DNA detection. Due to ethical and logistical constraints, our definition of OBI relied exclusively on peripheral blood testing. This methodological limitation may partly explain the lack of HBV DNA detection among atypical serological profiles in our cohort. In addition, excluding anti-HBs–positive samples might have overlooked immune escape mutants and the absence of genotyping and S-gene sequencing limited the detection of potential escape mutants.

Although the multivariate analysis did not reveal any factors independently associated with the atypical HBV profile, this absence of significant associations is likely due to limited statistical power related to the relatively small sample size. Larger studies with increased sample numbers are therefore needed to confirm these preliminary observations and to better identify independent predictors of atypical serological patterns.

### 4.2. Clinical Implications: Risk of Mother-to-Child Transmission and Recommendations for Diagnostic Improvement

Atypical HBV profiles in pregnant women may conceal low-level or intermittent viremia, posing a potential risk of mother-to-child transmission, especially during immune modulation in pregnancy or postpartum. To reduce this risk, we recommend targeted molecular testing for women with discordant serological markers, enhanced follow-up during pregnancy and after delivery, and the use of additional biomarkers such as HBV RNA or HBcrAg. Implementing these improvements could help optimize antiviral prophylaxis and immunoprophylaxis strategies, thereby minimizing the residual risk of vertical transmission. Strengthening molecular screening within local antenatal programs would also support Gabon’s efforts to align with WHO targets for HBV elimination by 2030.

## 5. Conclusions

This study highlights the significant occurrence of atypical hepatitis B serological profiles among HBsAg-negative women of childbearing age in Gabon, particularly among pregnant women. Although HBV DNA was not detected in our cohort, the observed profiles are suggestive of possible occult infections or immune escape variants that elude conventional screening. These findings emphasize the need to incorporate more sensitive molecular diagnostics alongside standard serology, particularly in prenatal care, to ensure early detection and reduce the risk of mother-to-child transmission. Enhanced surveillance and diagnostic refinement are essential to support public health efforts aimed at controlling hepatitis B infection in endemic and resource-constrained settings. Interestingly, these results highlight that pregnant women with isolated anti-HBc should be closely monitored, and antiviral prophylaxis may be considered in cases with detectable HBV DNA or additional risk factors for reactivation or vertical transmission.

## Figures and Tables

**Table 1:** Distribution of Enrolled Women According to Hepatitis B Serological Markers.

Markers					Pregnant		Non-Pregnant		Total	
HBsAg	Anti-HBs Ab	HBeAg	Anti-Hbe Ab	Anti-HBc Ab	n	(%)	n	(%)	N	(%)
										
-	-	-	-	-	29	(58, 0)	34	(68, 0)	63	(63, 0)
-	-	-	-		1	(2, 0)	0	(0, 0)	1	(1, 0)
-	-	-	+	-	8	(16, 0)	6	(12, 0)	14	(14, 0)
-	-	-	+	+	6	(12, 0)	3	(6, 0)	9	(9, 0)
-	-	-		-	1	(2, 0)	0	(0, 0)	1	(1, 0)
-	+	-	-	-	2	(4, 0)	2	(4, 0)	4	(4, 0)
-	+	-	+	-	1	(2, 0)	0	(0, 0)	1	(1, 0)
-	+	-	+	+	2	(4, 0)	4	(8, 0)	6	(6, 0)
-	-	+	-	-	0	(0, 0)	1	(2, 0)	1	(1, 0) *
Total					50	(100, 0)	50	(100, 0)	100	(100, 0)

* Profile potentially indicating occult hepatitis B infection.
**Legend**   :Negative :Positive :Blank = No reaction (invalid test)

## Data Availability

The original contributions presented in this study are included in the article. The datasets generated and/or analyzed during the current study are available from the corresponding author on reasonable request.

## Abbreviations

The following abbreviations are used in this manuscript:

HBV	Hepatitis B Virus
HBsAg	Hepatitis B Surface Antigen
Anti-HBs	Antibody to Hepatitis B Surface Antigen
HbeAg	Hepatitis B e Antigen
Anti-HBe	Antibody to Hepatitis B e Antigen
Anti-HBc	Antibody to Hepatitis B Core Antigen
OBI	Occult Hepatitis B Infection
CHUME-FJE	University Hospital Center Mother and Child Jeanne Ebori Foundation
RDT	Rapid Diagnostic Test
